# A Brief Music App to Address Pain in the Emergency Department: Prospective Study

**DOI:** 10.2196/18537

**Published:** 2020-05-20

**Authors:** Peter R Chai, Emily Schwartz, Mohammad Adrian Hasdianda, Desiree R Azizoddin, Anna Kikut, Guruprasad D Jambaulikar, Robert R Edwards, Edward W Boyer, Kristin L Schreiber

**Affiliations:** 1 Division of Medical Toxicology Department of Emergency Medicine Brigham and Women's Hospital Boston, MA United States; 2 Department of Psychosocial Oncology and Palliative Care Dana Farber Cancer Institute Boston, MA United States; 3 The Koch Institute for Integrated Cancer Research Massachusetts Institute of Technology Boston, MA United States; 4 The Fenway Institute Boston, MA United States; 5 Department of Anesthesiology, Perioperative, and Pain Medicine Brigham and Women's Hospital Boston, MA United States; 6 Department of Emergency Medicine Brigham and Women's Hospital Boston, MA United States

**Keywords:** music therapy, pain, smartphone, technology, telemedicine, emergency service, hospital

## Abstract

**Background:**

Emergency physicians face the challenge of relieving acute pain daily. While opioids are a potent treatment for pain, the opioid epidemic has ignited a search for nonopioid analgesic alternatives that may decrease the dose or duration of opioid exposure. While behavioral therapies and complementary medicine are effective, they are difficult to deploy in the emergency department. Music is a potential adjunctive therapy that has demonstrated effectiveness in managing pain.

**Objective:**

Our objective was to understand the feasibility and potential for an effect of a novel music app to address acute pain and anxiety in patients admitted to an emergency department observation unit.

**Methods:**

This prospective cohort study enrolled patients admitted to an emergency department observation unit with pain who had received orders for opioids. We gathered baseline pain and psychosocial characteristics including anxiety, sleep disturbance, and pain catastrophizing using validated questionnaires. Participants received a smartphone-based music intervention and listened to the music in either a supervised (research assistant–delivered music session 3 times during their stay) or unsupervised manner (music used ad lib by participant). The app collected premusic and postmusic pain and anxiety scores, and participants provided qualitative feedback regarding acceptability of operating the music intervention.

**Results:**

We enrolled 81 participants and randomly assigned 38 to an unsupervised and 43 to a supervised group. Mean pain in both groups was 6.1 (1.8) out of a possible score of 10. A total of 43 (53%) reported previous use of music apps at home. We observed an overall modest but significant decrease in pain (mean difference –0.81, 95% CI –0.45 to –1.16) and anxiety (mean difference –0.72, 95% CI –0.33 to –1.12) after music sessions. Reduction of pain and anxiety varied substantially among participants. Individuals with higher baseline pain, catastrophizing (about pain), or anxiety reported greater relief. Changes in pain were correlated to changes in anxiety (Pearson ρ=0.3, *P*=.02) but did not vary between supervised and unsupervised groups. Upon conclusion of the study, 46/62 (74%) reported they liked the music intervention, 57/62 (92%) reported the app was easy to use, and 49/62 (79%) reported they would be willing to use the music intervention at home.

**Conclusions:**

A smartphone-based music intervention decreased pain and anxiety among patients in an emergency department observation unit, with no difference between supervised and unsupervised use. Individuals reporting the greatest reduction in pain after music sessions included those scoring highest on baseline assessment of catastrophic thinking, suggesting there may be specific patient populations that may benefit more from using music as an analgesic adjunct in the emergency department. Qualitative feedback suggested that this intervention was feasible and acceptable by emergency department patients.

## Introduction

### Background

The number of opioid analgesics prescribed in the United States is now recognized as unsustainably and dangerously high [1,2], with no indication that the experience of pain among individuals has improved as a result. Three problematic consequences of increased opioid prescription are (1) overdose deaths, with over 47,000 individuals dying from opioid-related overdose in 2017 [3] (approximately 128 individuals per day); (2) an epidemic of opioid use disorder, with escalation to injection opioid use and increasing numbers of opioid-related bacterial infections [4]; and (3) an inherent conflict of clinical priorities in the emergency department (ED), where providers confront the competing pressures of reducing exposure to opioid analgesics while still managing acutely painful conditions, with limited alternatives [5,6]. Nonopioid analgesics, increasingly the first-line treatment for certain acutely painful conditions, often provide an inadequate response. Enhancing the response to nonopioid (or opioid) analgesics through adjunctive use of behavior modulatory therapies (eg, cognitive behavioral therapy) [7] or complementary medicine (eg, yoga, acupuncture) is an attractive strategy [8,9], but these therapies are difficult to deploy in the ED. Therefore, exploration of other more feasible and scalable alternative strategies is needed to reduce or replace opioid pharmacotherapy in this context.

One potential adjunctive therapy is listening to music, a nearly universal experience shared across cultures, ethnicities, and races [10]. The ubiquity of music across cultures and known human history may arise from its capacity to improve affect and mood. Functional magnetic resonance imaging studies have shown that pleasurable music listening is associated with increased dopaminergic neurotransmission in the nucleus accumbens, a common reward pathway in the brain [11,12]. Music also improves affect, with at least one study showing a beneficial effect on both pain and anxiety in the perioperative setting [13].

The favorable neurochemistry induced by listening to music may enable an innovative behavioral intervention. Machine learning has identified features (ie, musical “genes”) associated with increased dopaminergic neurotransmission and improved affect while listening to pleasurable music [14,15]. These features intrinsic to music itself have formed the basis for commercial music selection algorithms such as the music genome project [16]. Similar techniques have been applied in the development of novel music programs to create new signatures of pleasurable music to be played from a smartphone app [17,18].

### Objective

The objective of this study was to evaluate the feasibility and effectiveness of a smartphone-based music intervention for acute pain in patients presenting to the ED. We specifically sought to measure changes in pain and anxiety associated with 10-minute music listening sessions delivered in either a supervised or an ad hoc fashion to patients with pain symptoms in an ED observation unit.

## Methods

### Participant Recruitment

In this prospective study, we recruited patients who were admitted to an ED observation unit in a quaternary, urban, level I trauma academic ED. Study staff identified potential participants by screening the electronic medical record and liaising with ED clinical staff. English-speaking patients with ED observation unit stays of up to 48 hours and who had received orders for opioid analgesia were eligible to participate. We excluded patients who had a pacemaker, had hearing loss, were on contact precautions, had previously enrolled in the study, were unstably housed, or had a significant active medical or psychiatric illness. A trained research assistant (RA) explained the study in full and gauged potential participants’ interest in the study. Interested participants provided verbal consent. The Partners Human Research Committee (Institutional Review Board of Partners HealthCare, Boston, MA, USA) approved this protocol.

Participants provided basic demographic information and underwent baseline pain and psychosocial assessments using validated measures (see section on measures) using a study phone with direct connection to a REDCap data capture system version 9.5 (REDCap Consortium) [19]. Next, participants were introduced to and given instructions for operating the novel music app on a study smartphone (iPhone 6; Apple Inc, Cupertino, CA, USA). We were interested in 2 modalities of use of the music app. First, we wanted to understand the use of music by participants if they were not given formal guidance as to how often and how long they should use music for. Second, we wanted to determine the feasibility of using a regimented prescription of music supervised by an RA. We therefore randomly assigned participants via a predetermined, computer-generated random list into unsupervised versus supervised intervention use groups. Participants randomly assigned to the unsupervised group received a smartphone with the app preloaded and paired noise-canceling headphones and were encouraged to use the music intervention app on an as-needed basis. Participants also received contact information for RAs so they could seek help to troubleshoot any potential issues with the app or the devices used. For participants randomly assigned to the supervised intervention group, a study RA reminded and assisted the participant to use the music app up to 3 times during their ED observation unit stay (approximately every 4 hours).

### Measures

We used several measures to assess baseline patient characteristics.

#### Pain

The Brief Pain Inventory (BPI) is a 9-item, self-report, validated measure that assesses patients’ pain severity (least, worst, and average) and functional interference in the preceding week using an 11-point Likert scale with higher scores indicating greater pain or functional impact, or both [20].

#### Psychosocial Characteristics

We used the Patient-Reported Outcomes Measurement Information System (PROMIS) depression (8 items), sleep disturbance (8 items), and anxiety (7 items) short forms [21]. All PROMIS measures were scored on a 5-point Likert scale with higher scores indicating higher symptomatology. The Pain Catastrophizing Scale (PCS) comprises 13 items exploring individuals’ pain catastrophizing divided into 3 subscales for rumination about pain, magnification of pain, and perceived helplessness to do anything about pain [22]. The PCS uses a 5-point Likert scale with cumulative scores ranging from 0 to 52. The Perceived Stress Scale consists of 10 items to measure subjective stress and uses a 5-point Likert scale for each item, where higher total scores indicate greater stress (range 0-40) [23]. We measured somatization using the 7-item somatization subscale from the Brief Symptom Inventory, where response options were scored on a 5-point Likert scale, with higher scores indicating greater somatization [24].

### The Music Intervention

Participants interacted with a smartphone-based music app that used 5 separate commissioned instrumental tracks intended to be relaxing (Unwind; Bose Corporation, Boston, MA, USA) [25]. These music tracks were composed using a backbone of musical genes derived through a machine learning algorithm that selected key musical features expected to induce subjective “thrills” among individuals, combined with human-composed music. Users were presented with a preview screen that allowed them to sample 5 different tracks and select their favorite track. The same 5 tracks were presented to each participant. Next, participants used noise-canceling headphones to listen to 10 minutes of the selected track. Participants rated their *pain* and *anxiety* using a 10-point Likert scale at the beginning and end of the 10-minute music session. At completion of the study procedures, participants were guided through an RA-administered survey to assess the feasibility of operating the music app and formative reflections on using music in the ED observation unit. We collected data on the total opioid administered during the participants’ ED observation unit stay from the electronic medical record and converted does to milligram morphine equivalents (MMEs). We then divided the MME amount by the number of hours the patient spent in the ED observation unit to calculate a normalized opioid utilization score (MME/h) for each participant during the study.

### Data Analysis

We summarized participant characteristics using frequencies and percentages for categorical variables, and mean and standard deviation or median and interquartile range for continuous variables, according to normality of distribution. For patient-reported scores of baseline and postmusic session pain and anxiety, we calculated mean values for each patient. We compared mean premusic versus mean postmusic scores using a nonparametric paired test (related-samples Wilcoxon signed rank test). We averaged the change in pain scores by subtracting mean end pain score from mean beginning pain score; we assessed this value (change in pain with music) for correlation with baseline patient characteristics, including psychosocial (anxiety, depression, catastrophizing, stress) and general pain severity and interference (BPI) questionnaires, using Spearman or Pearson correlations, as appropriate. All statistical tests were 2-tailed, and level of significance was set at Cronbach α=.05. We performed all analyses using IBM SPSS 25 (IBM Corporation).

## Results

### Participant Characteristics

**Figure 1:**
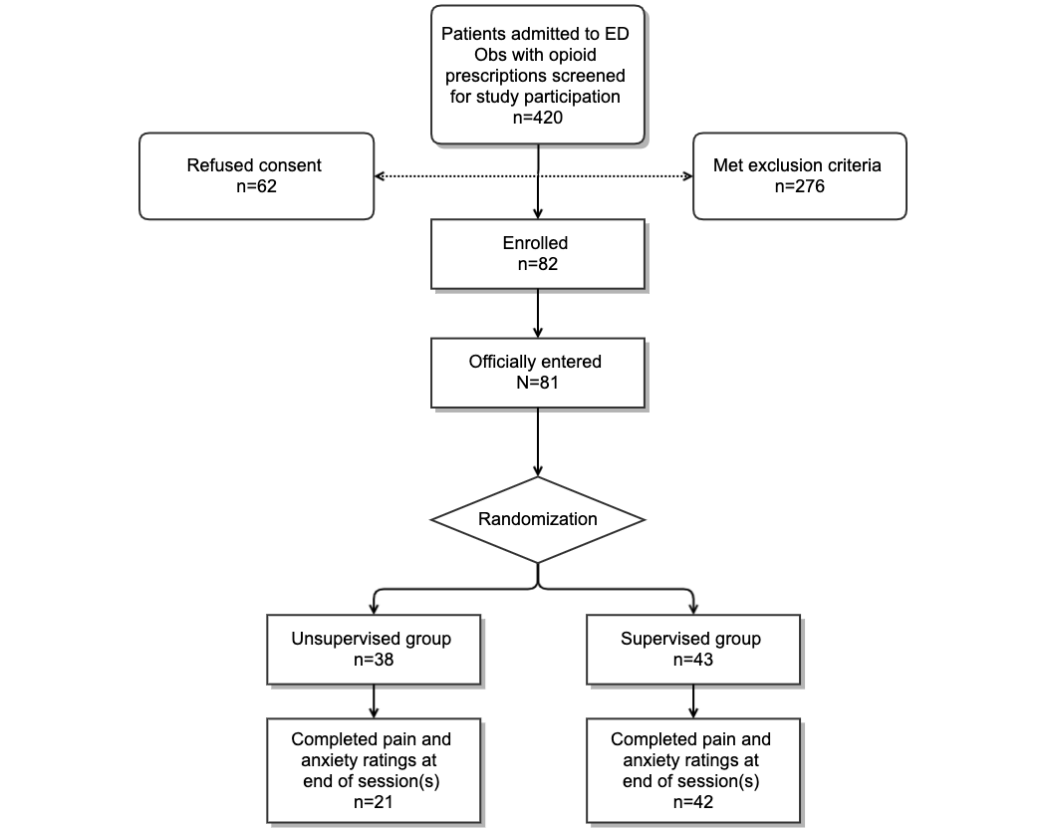
Study flow diagram. ED Obs: emergency department observation unit.

We screened 420 individuals, of whom 144 were eligible for participation (Figure 1). Of these, we enrolled 82 patients. Common reasons for nonparticipation were lack of interest in research, other concomitant ongoing clinical assessments, and the inability to operate a smartphone.

The mean age of participants in the total sample (N=81) was 43.84 (SD 15) years, with a larger proportion of men (57, 70%) than women (24, 30%; Table 1). Reported pain in the previous week was high (BPI mean pain score 6.12, SD 1.8, out of 10), and at baseline 14 (17%) of participants were taking opioids on a regular basis. Participants reported relatively elevated scores on depression, anxiety, stress, and somatization (Table 1). Similarly, pain catastrophizing scores were higher than in previous reports of healthy volunteers [22] and similar to ranges reported among patients with chronic pain. We identified no difference between the supervised and unsupervised groups in terms of baseline pain or psychosocial characteristics (*P*>.05).

**Table 1:** Patient sample characteristics (N=81).

Characteristics		Values
Age (years), mean (SD)		43.84 (15)
Female sex, n (%)		24 (30)
Taking opioids at baseline, n (%)		14 (17)
**Baseline Brief Pain Inventory (BPI) score, mean (SD)**		
	BPI current	6.17 (2.1)
	BPI worst	8.61 (2.2)
	BPI least	3.02 (2.4)
	BPI mean	6.12 (1.8)
	BPI interference	6.22 (2.4)
**Patient-Reported Outcomes Measurement Information System score, mean (SD)**		
	Anxiety (score range 7-35)	19.13 (6.2)
	Depression (score range 8-40)	17.38 (7.5)
	Sleep Disturbance (score range 8-40)	29.22 (6.4)
Somatization (Brief Symptom Inventory; score range 0-35), mean (SD)		12.61 (4.0)
Perceived Stress Scale (score range 0-40)		18.83 (3.5)
**Pain Catastrophizing Scale (score range 0-52), mean (SD)**		
	Rumination	8.35 (4.7)
	Magnification	3.28 (2.5)
	Helplessness	10.48 (6.4)
	Total	22.11 (12.5)

### Change in Pain During a Music Session

**Figure 2:**
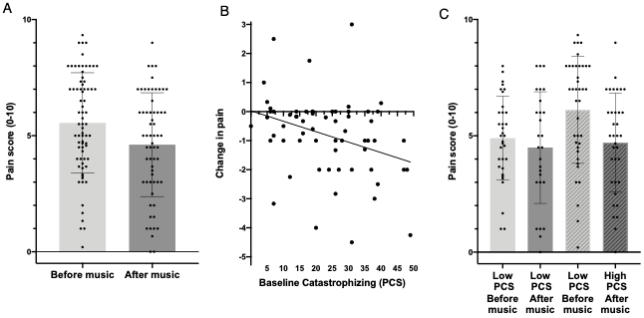
Effect of music on pain in emergency department patients. (A) Comparison of mean pain scores before and after music interventions revealed a significant overall reduction in pain scores in most individuals (related-samples Wilcoxon signed rank test, *P*<.001). The mean change in pain score with music was modest (mean difference –0.81, 95% CI –0.45 to –1.16) with considerable variability among participants. (B) Baseline Pain Catastrophizing Scale (PCS) score measured before music interventions was correlated with the amount of change in pain after music session. Higher baseline PCS scores were associated with a larger decrease in pain after music session (Spearman ρ=–0.39, *P*=.009). (C) Change in pain after music among low and high catastrophizers. Patients with high baseline PCS score (>20) had a greater decrease in pain (mean difference –1.2, SD 1.4) after music session than those with lower PCS (mean difference –0.3, SD 1.1; independent-samples *t* test=–2.9, *P*=.005).

A total of 73 participants successfully attempted the music sessions, with a mean of 3 music sessions and a range of 1 to 9 music sessions per participant. We obtained fewer postmusic pain ratings than premusic ratings, with the missed postmusic ratings frequently being due to the participant falling asleep while listening to the music, as noted by RAs in supervised administrations. Comparison of mean pain scores before and after music interventions revealed a significant overall reduction in pain scores in most individuals (Figure 2, part A; related-samples Wilcoxon signed rank test, *P*<.001). The mean change in pain scores with music was modest (mean difference –0.81, 95% CI –0.45 to –1.16), with considerable variability among participants. To explore participant characteristics associated with the greatest benefit, we correlated the magnitude change in pain scores to baseline characteristics. Those with higher current pain reported on the BPI experienced greater pain relief with the use of music (Spearman ρ=–0.37, *P*=.004).

### Patient Characteristics Associated With Pain Reduction

The psychosocial variables anxiety, depression, sleep disturbance, and catastrophizing showed significant intercorrelation to each other, as well as a significant association with reported acute pain (Multimedia Appendix 1). The PCS score at baseline was inversely correlated with the change in pain during the music session (difference between the end and beginning pain scores; (Spearman ρ=–0.39, *P*=.009; Figure 2, part B); participants with initial PCS scores greater than 20 were more likely to report a significant decrease in pain with the music intervention (Figure 2, part C). Other psychosocial variables (anxiety, depression, somatization, stress) were not significantly related to the degree of music-induced change in pain. Pain reduction did not differ between the supervised and unsupervised groups (*P*>.05).

### Change in Anxiety During a Music Session

**Figure 3:**
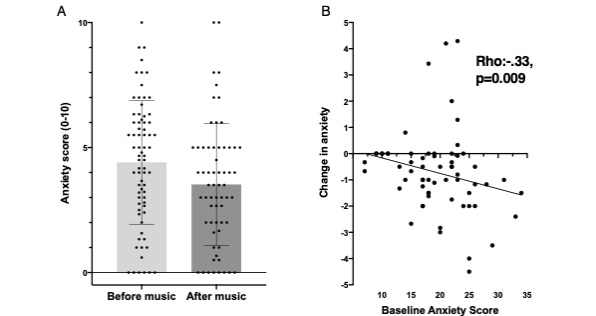
Effect of music on anxiety in emergency department patients. (A) Comparison of mean anxiety scores reported before and after the music listening intervention revealed a significant overall reduction in anxiety among individuals (related-samples Wilcoxon signed rank test, *P*<.001). The mean magnitude change in anxiety score with music was modest (mean difference –0.72, 95% CI –0.33 to –1.12) with considerable variability among participants. (B) Higher baseline anxiety scores were associated with a greater reduction in anxiety scores before and after the music session (Spearman ρ=0.3, *P*=.02).

Comparison of mean anxiety scores reported before and after the music intervention revealed a significant overall reduction in anxiety in most individuals (Figure 3, part A; related-samples Wilcoxon signed rank test, *P*<.001). As with pain, the mean magnitude of change in anxiety scores with music was modest (mean difference –0.72, 95%CI –0.33 to –1.12), with considerable variability among participants. Greater reduction in anxiety scores significantly correlated with baseline anxiety, with those reporting high baseline anxiety also showing a greater reduction in anxiety with music (Figure 3, part B). Other psychosocial variables (depression, somatization, stress, catastrophizing) were not significantly related to the degree of music-induced change in anxiety. Change in pain was modestly related to change in anxiety (Pearson ρ=0.30, *P*=.02). Anxiety reduction did not differ between the supervised and unsupervised groups (*P*>.05).

### Relationship of Baseline Characteristics to Opioid Use While in the ED Observation Unit

**Figure 4:**
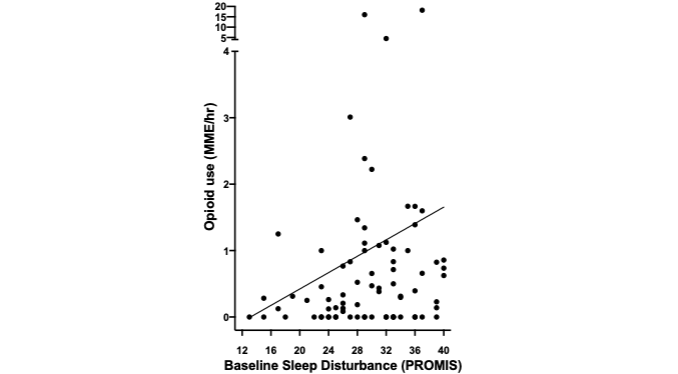
Variation in opioid use among participants with baseline sleep disturbance. Baseline sleep disturbance (measured by the Patient-Reported Outcomes Measurement Information System [PROMIS] short form) was associated with a higher amount of opioid consumption (milligram morphine equivalent [MME]) per hour while in the emergency department (Spearman ρ=0.24, *P*=.03).

As a secondary exploratory analysis, we examined whether certain patient characteristics were associated with higher opioid use during the ED observation unit stay. A higher reported baseline sleep disturbance was associated with increased opioid consumption (Figure 4). Other baseline patient characteristics were not significantly related to changes in opioid use.

### Feasibility and User Experience

Analyses of the exit survey data revealed that most participants had used music as therapy before (43/62, 69%), and many participants had used other music apps in the past (23/62, 37%). The majority of participants reported they liked the music app (46/62, 74%), with the majority (43/62, 69%) saying they used it to relax during their ED stay, and some (15/62, 24%) endorsed using the music to sleep (Table 2). Most participants (49/62, 79%) said they would use it at home.

**Table 2:** Summary of the exit survey impressions of the Unwind App (n=62).

User experience	Values, n (%)
App was easy to use	57 (92%)
Liked the music app	46 (74%)
Purpose of app use: relax	43 (69%)
Purpose of app use: sleep	15 (24%)
Would use the app at home	49 (79%)

## Discussion

### Principal Findings

These findings suggest that music may be used to decrease pain and anxiety among individuals admitted to an ED observation unit. Individuals who had increasing catastrophizing at baseline (measured by the PCS) experienced greater decreases in pain and anxiety after listening to the music app. This demonstrates that music interventions may potentially be applied with greater effect in individuals with higher catastrophizing in the ED. Our findings are consistent with other investigations that described many other sources of music to be associated with the reduction of postoperative or procedural pain in individuals with chronic pain [26-32]. Additionally, the feasibility of using this music intervention in both a supervised and an unsupervised model suggests that this intervention can be used as a prescribed, targeted intervention in the ED or used ad hoc in individuals who may experience pain outside of the ED.

Music has several hypothesized effects on pain. First, music may modulate affect by impacting reward pathways, as changes in dopaminergic neurotransmission are associated with pleasurable or relaxing music. Music may decrease other pain-augmenting factors such as anxiety. Second, music may alter the nociceptive processing of pain by changing pain tolerance and threshold [33-35]. Alternatively, music may divert attention from acute pain to a different, pleasurable stimulus [10,18,30-33,35-38]. Regardless of the mechanism, the universality of music—every culture on the globe has some form of musical expression—coupled with the near ubiquity of personal music players, smartphones, and streaming music services makes music an attractive potential adjunctive therapy for pain.

Our data suggest that this app-based, semistructured music intervention can be employed during brief ED observation unit admissions to reduce pain and anxiety in patients with acute pain. Participants reported pain scores in real time and operated a music app in both supervised and unsupervised conditions. Additionally, participants’ pain and anxiety scores were not different between the supervised and unsupervised groups. Integrated into emergency care, the use of music may afford an adjunctive treatment to those patients with higher catastrophizing and anxiety, which may be particularly relevant in emergency care settings [5].

While music reduces pain and anxiety in controlled settings [26-32], our investigation demonstrated that these effects may also exist in the midst of the unpredictable environment of the ED. These decreases in pain and anxiety, while modest, may meaningfully alter the ED experience for some individuals by serving as an adjunct to opioid analgesics. While this study was not designed to evaluate the effect of music on opioid consumption, its potential combination with other nonopioid interventions could contribute to decreased opioid use while in the ED and decrease opioid dosage units prescribed at discharge [10].

Notably, the modulation of pain produced by this music intervention was greatest in individuals with high levels of catastrophizing (negative cognitions and rumination about pain). Similarly, reduction of anxiety during the intervention was largest among those with highest baseline anxiety levels. The finding that music has a greater impact on those individuals with high levels of baseline catastrophizing anxiety is notable, as these traits are often associated with greater resistance to pharmacological manipulation, as well as pain persistence. Other previous studies among pain patients have found an association between greater catastrophizing scores and greater efficacy of behavioral interventions directed at affect, mood, and catastrophizing [39-41]. Whether the pain-sparing effect of music among high catastrophizers is due to alteration of sensory perception of nociceptive stimuli, as some laboratory-based investigations have suggested [17,18,28], or whether music modulates affect and thus indirectly decreases the impact of painful stimuli [27,36] remains to be clearly defined. Attentional modulation of (distraction away from) painful stimuli may be an important aspect of how music provides pain relief [41], although it seems unlikely to be the sole mechanism, as distraction alone has not led to pain reductions in similar populations [42].

Our exit survey data of patients at the end of the study support a possible role of affect modulation in music’s effect. For example, one participant described that they continued to experience pain while listening to the music but felt “better” about their pain. Others reported decreased anxiety about their pain, while some notably reported that they preferred the use of music over receiving an opioid. Interestingly, one observation from the study was that many participants fell asleep during the listening session, consistent with many previous studies that have noted an important connection between sleep deprivation and pain [43]. These findings suggest that the interplay between music and pain is complex. We anticipate that future investigations will address both the mechanisms in which music therapy may integrate within the biopsychosocial model of pain, and the implementation science behind operationalizing a recommendation for music to address the experience of pain.

### Limitations

This study had several limitations. First, we conducted our study at a single urban academic ED. Experiences regarding complaint and psychosocial profiles of study participants and the resources available in ED observation units may be different in other types of ED settings, thus limiting generalizability to similar cohorts. Second, participants operating the app did not always rate postintervention pain and anxiety each time they used it. Anecdotally, participants reported that listening to the music made them fall asleep—itself a highly beneficial effect—thus leaving them unable to complete the postassessment, and possibly underestimating the degree of pain and anxiety reduction. This, taken together with the observation that individuals with the greatest sleep disturbance used the most opioids, suggests that future work should explore the use of music intervention to augment sleep in individuals with pain. Third, because our primary objective was the effectiveness and feasibility of using music to reduce pain and anxiety, we employed a pre-post assessment over multiple sessions, and there was thus not a control group in this study. Fourth, the use of the music app was not directly tied to opioid administration in the ED observation unit. We did not encourage participants or nursing staff to access the music app at particular times proximal to their scheduled opioid administration. Future investigations should consider offering music in strategic times near opioid administration to understand whether the analgesic effects of music can diminish the times individuals request opioids. This also made it impossible to investigate the impact of music on opioid use during the study period.

### Conclusion

A smartphone-based intervention built on machine learning–based musical knowledge produced a significant decrease in both pain and anxiety among ED observation unit patients with acute pain. Participants experienced decreased pain and anxiety regardless of whether they used the music intervention on an as-needed basis for pain or under direct supervision. Individuals with higher catastrophizing scores at baseline gained more benefit from the music intervention. Further randomized, controlled studies evaluating the effect of different types of music intervention on pain modulation, pain catastrophizing, and opioid use are needed [43]. Careful assessment of baseline characteristics may help further delineate those who are most likely to derive analgesic benefit from music. Ultimately, our results demonstrate that a music intervention is a feasible adjunctive therapy for individuals who have pain in the ED, as participants willingly used a music intervention when offered to them.

## Appendix

Multimedia Appendix 1 Correlation table between psychosocial factors and pain.

## Abbreviations

BPI: Brief Pain Inventory
ED: emergency department
MME: milligram morphine equivalent
PCS: Pain Catastrophizing Scale
PROMIS: Patient-Reported Outcomes Measurement Information System
RA: research assistant
