# Supplementary figures and images for: A Brief Music App to Address Pain in the Emergency Department: Prospective Study
Source: J Med Internet Res. 2020 May 20;22(5):e18537. doi: 10.2196/18537 (PMC7270860; doi:10.2196/18537)

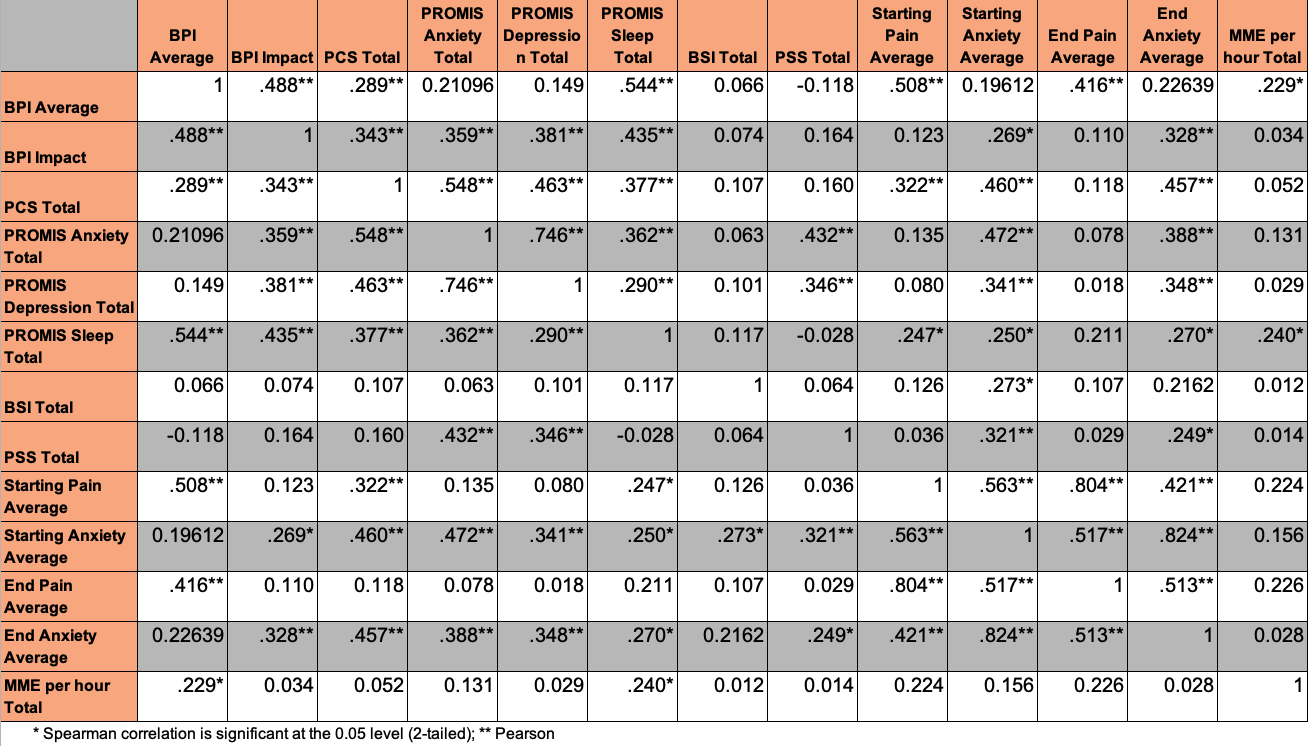

Supplement: Multimedia Appendix 1 [file jmir_v22i5e18537_app1.png]
